# Soy Protein Isolate Gel Subjected to Freezing Treatment: Influence of Methylcellulose and Sodium Hexametaphosphate on Gel Stability, Texture and Structure

**DOI:** 10.3390/foods13132117

**Published:** 2024-07-02

**Authors:** Xiaoyu Xia, Binyang Zhang, Yuyang Huang, Ying Zhu, Min Qu, Linlin Liu, Bingyu Sun, Xiuqing Zhu

**Affiliations:** College of Food Engineering, Harbin University of Commerce, Harbin 150028, China; 18104568558@163.com (X.X.); z983046926@163.com (B.Z.); huangyuyang1979@163.com (Y.H.); 13258512068@163.com (Y.Z.); qumin777@126.com (M.Q.); keaiduolinlin@126.com (L.L.); sby0451@163.com (B.S.)

**Keywords:** soy protein gel, methyl cellulose, sodium hexametaphosphate, freezing

## Abstract

Freezing affects texture and induces the loss of gel quality. This study investigated the effects of methylcellulose (MC) (0.2%, 0.4%, 0.6%) and sodium hexametaphosphate (SHMP) (0.15%, 0.3%) on the gel textural and structural properties of SPI gels before and after freezing, and explores the synergistic enhancement of gel texture and the underlying mechanisms resulting from the simultaneous addition of SHMP and MC to SPI gels. It was revealed that MC improved the strength of SPI gels through its thickening properties, but it could not inhibit the reduction of SPI gels after freezing. The 0.4% MC-SPI gel exhibited the best gel strength (193.2 ± 2.4 g). SHMP inhibited gel reduction during freezing through hydrogen bonding and ionic interactions; it enhanced the freezing stability of SPI gels. The addition of 0.15% SHMP made the water-holding capacity in SPI gels reach the highest score after freezing (58.2 ± 0.32%). The synergistic effect of MC and SHMP could improve the strength and the freezing stability of SPI gels. MC facilitated the release of ionizable groups within SPI, causing negatively charged SHMP groups to aggregate on the SPI and inhibit the freezing aggregation of proteins. These results provide a strong basis for the improvement of cryogenic soy protein gel performance by SHMP and MC.

## 1. Introduction

In recent years, increasing attention has been directed towards plant proteins [1], with particular focus on soy protein isolate (SPI) due to its exceptional nutritional value and strong gelation properties. The texture of soy-based foods is significantly dependent on the gelation properties of soy proteins [2]. However, pure SPI gels exhibit deficiencies in terms of mechanical properties and gel strength. Many studies have addressed these issues by introducing polysaccharides to interact with protein molecules, leading to significant improvements in gel structure and functional characteristics [3]. Zhang et al. [4], for instance, introduced nanocrystals into SPI gels, which resulted in denser and more uniform gel networks. Gan et al. [5] utilized xanthan gum to induce gel formation in SPI through heat, which resulted in significantly higher gel strength compared to pure SPI systems. Methylcellulose (MC), a non-ionic cellulose ether with excellent thickening properties, can interact with soy proteins, thereby creating a robust gel network structure that yields denser and more uniform gels [6]. However, composite SPI gels formed with the addition of MC still face challenges such as susceptibility to syneresis and poor water retention [7].

Freezing treatment is a vital food preservation method. During freezing, the formation of ice crystals results in gel dehydration and a reduction in texture. Low-density ice crystals formed during phase transition disrupt the protein gel network [8], especially the water migration caused by freezing [9]; ice crystal formation increases the proportion of free water in the gel, leading to an irreversible reduction [10]. In comparison to polysaccharides, the effect of phosphates on improving SPI gel strength is less pronounced [2], with ternary composite gels with stronger interactions being widely considered due to their superior mechanical properties. Majid et al. [11] suggested that protein–polysaccharide/salt composite gels possess complementary mechanisms. The addition of salt ions to SPI gels can enhance the freezing stability and mechanical properties of the gel [12]. Sodium hexametaphosphate (SHMP) is an excellent food humectant, containing phosphate ions that can chelate metal cations in proteins, release hydrophilic carboxyl groups, facilitate water absorption, and effectively inhibit ice crystal formation [13]. Jin et al. [14] also suggested that phosphates promote the absorption of water by proteins, thereby improving the water-holding capacity of protein gels. Based on existing findings, to date, reports on the improvement of gel freezing stability through protein–polysaccharide/salt composite gels are still limited.

This study evaluated the influence of MC and SHMP on the structure and texture of frozen SPI gels, as well as investigated the interactions and mechanisms between MC and SHMP. We believe that the comprehensive synergistic effect of MC and SHMP may not only form a dense and uniform gel network structure, but also reduce the reduction effect of freezing treatment on gel. This research provides a promising solution for the application of SPI in frozen food products.

## 2. Materials and Methods

### 2.1. Materials

SPI with 91.2% protein, 0.53% carbohydrate and 0.65% fat (dry basis) were purchased from Jinlong Co., Ltd., (Harbin, China). Methylcellulose (MC) with a viscosity of 400 mPa·s was purchased from Damao Chemical Reagent Factory (Tianjin, China). Sodium hexametaphosphate (SHMP) was purchased from Jinko Chemical Co., Ltd., (Wuxi, China). All chemical reagents were purchased from Tianjin Tianli Chemical Reagent Co., (Tianjin, China) and were of analytical grade.

### 2.2. Preparation of SPI Gel

According to the method of [3], SPI was slightly modified by adding it to deionized water (protein mass fraction of 14%), followed by stirring at room temperature for 60 min at 600× *g* using a magnetic stirrer (Ultra-Turrax T18 model, Ika Co., Ltd., Berlin, Germany). SHMP (control, 0.2% MC, 0.4% MC, 0.6% MC, 0.15% SHMP, 0.3% SHMP, 0.2% MC + 0.15% SHMP, 0.4% MC + 0.15% SHMP, 0.6% MC + 0.15% SHMP, 0.2% MC + 0.3% SHMP, 0.4% MC + 0.3% SHMP, 0.6% MC + 0.3% SHMP) were added to the SPI solution, respectively, and then stirred for another 60 min to ensure uniform distribution of each component. Samples were sealed and kept overnight at 4 °C to ensure complete protein hydration. Each SPI sample (20 mL) was placed in a glass vial with an internal diameter of 40 mm and bathed in water at 90 °C for 30 min using a thermostatic water bath to heat (HH-4, Jiangsu Jintan Honghua Co., Ltd., Chengdu, China). The heated samples were immediately cooled to normal temperature in an ice bath; a portion was taken for viscoelasticity analyses and the other stored in a sealed refrigerator at 4 °C for 24 h before use.

### 2.3. Treatment by Freezing

SPI gel samples were frozen at −18 °C for 45 h and thawed at 25 °C for 3 h. The obtained samples were freeze-dried using a freeze dryer (ALPHA1-2LD plus model, Christ Co., Ltd., Berlin, Germany), ground into powder at room temperature and passed through an 80-mesh screen. All freeze-dried samples were stored in airtight containers in a 4 °C freezer for subsequent analysis.

### 2.4. Water-Holding Capacity

The water-holding capacity of the gel samples was determined according to the method described by Chen [2], with slight modifications. Gel (2 g, W_1_) was centrifuged at 10,000× *g* and 4 °C for 20 min using a frozen centrifuge (GL21M model, Xiangyi Co., Ltd., Shijiazhuang, China). The gel was then removed, the surface moisture was drained using filter paper, and weighed again (W_2_). The mass of the gel sample after centrifugation was recorded and the water-holding capacity of the gel was calculated according to the following formula. The moisture content indicates the water-holding capacity of the samples.

$$WHC\;(\%)=\frac{W_{2}}{W_{1}}\times 100$$

### 2.5. Texture Properties (TPA)

Texture profile analysis of the gel samples was achieved using a modified method of Jin et al. [14]. Texture profile analysis (TPA) is the most commonly used method, which utilizes a two-cycle uniaxial compression test to characterize texture features that include hardness, springiness, adhesiveness and chewiness [15]. Gel samples were formed into a cylindrical shape (10 × 20 mm, R × H) using a texture analyzer (TA. XT Plus, Stable Micro System Co., Ltd., New York, NY, USA) and the gel textural properties were determined. The test parameters were as follows: 5 mm/s pre-test speed; 1 mm/s test speed; 5 mm/s post-test speed; 40 mm test distance; 50% compression deformation; and 5 g trigger force. Each sample was analyzed 3 times.

### 2.6. Viscoelasticity Analyses

Based on the method of Huang et al. [13], the viscoelastic properties of the gel samples were measured using an advanced rotary rheometer (MCR-102, Antongpa Trading Co., Ltd., Shanghai, China). The rheometer was equipped with a bottom plate and parallel plate, both with a diameter of 60 mm and a fixed spacing of 1 mm. The SPI concentration in the gel sample was 14% (*w*/*v*). Sample gels were loaded on the bottom plate and were covered with a lid to limit evaporation and mitigate external noise and temperature interference. To prevent water evaporation, a thin layer of silicone oil was applied to the edges of the lid. Before each measurement, the sample was balanced on a parallel plate at 25 °C for 2 min. The frequency sweep test was conducted in the range of 0.1–10 rad/s with a strain of 0.5% which was within the linear viscoelastic region of the gels utilized. Storage modulus (G’) and the loss modulus (G”) were used.

### 2.7. Low-Field Nuclear Magnetic Resonance (LF-NMR)

LF-NMR analysis of the samples extracted from the gels (1 g) was carried out using the method described by Zhang et al. [4], with some slight modifications. Samples were placed in a 7 mm NMR tube and covered to prevent water loss. T_2_ values were measured using a Carr–Purcell–Meiboom–Gill pulse train with 12,288 echoes (5 data points per echo) on an Ultra NMR spectrometer (Niumag Co., Ltd., Shanghai, China) at a magnetic field of 0.72 T and 1 H resonance frequency of 30.7 MHz. Data were analyzed using RINMR software (NMI v20 Suzhou Niumai Electronic Technology Co., LTD., Suzhou, China). The sampling time between the data points of each echo was 10 μs, resulting in a spectrum width of 100 kHz. The interval time of each echo was 407 μs. A total of 16 transients were recorded, with no baseline shift in the phase cycle and a repeat time of 30 s used. Each CPMG echo sequence was averaged to one data point using the IDL package (ITT Visual Information Solutions, Boulder, CO, USA). Experimental errors (upper and lower limits) were determined by repeating all experiments three times.

### 2.8. Intermolecular Force

The intermolecular force in the gel samples was determined according to the method of Tanger et al. [16], which was slightly modified. Using different denaturing solvents to treat samples can disrupt different intermolecular forces. In fact, 0.6 mol/L NaCl can break ionic bonds; 1.5 mol/L urea can break hydrogen bonds; 8 mol/L urea can disrupt hydrogen bonds and hydrophobic interactions; and 1.5 mol/L β-mercaptoethanol can break disulfide bonds. Extraction solvent preparation: S1: 0.6 mol/L NaCl; S2: 0.6 mol/L NaCl + 1.5 mol/L urea; S3: 0.6 mol/L NaCl + 8 mol/L urea; and S4: 0.6 mol/L NaCl + 8mol/L urea + 1.5 mol/L β-mercaptoethanol. Sample extraction and dilution: Gel samples were freeze-dried and crushed through an 80-mesh screen. Sample powder (0.25 g) was inserted in a 50 mL centrifuge tube and 5 mL of the above four solvents were mixed thoroughly, extracted at 4 °C for 5 h, centrifuged (10,000 r/min, 10 min) and the supernatant was stored at 4 °C for use. Five milliliters of the above four solvents were added to the precipitation, the extraction was repeated twice, then centrifuged. Protein content in the supernatant was determined by the Coomathi bright blue method (determination wavelength 595 nm) using a UV–Vis spectrophotometer (ALPHA 1650, Shanghai, China). The following chemical bond representation method (protein content) was employed: Ionic bond: S1; Hydrogen bond: S2–S1; Hydrophobic interaction: S3–S2; and Disulfide bond: S4–S3.

### 2.9. Fourier Transform Infrared (FTIR)

Proper modifications were performed following the method of Tang et al. [17], and FTIR spectra were recorded on a Spectrum Two infrared spectrometer (PerkinElmer, Shelton, CT, USA). The gel freeze-dried powder was dried thoroughly using P_2_O_5_ in a dryer and placed directly on the germanium crystal surface of a horizontal ATR instrument. After tablet pressing, the sample plate required for the FTIR experiment was obtained. A total of 32 scans with absorption spectra measured in the range of 4000–400 cm^−1^ and the background spectrum scanned with a resolution of 4 cm^−1^ were used. The relative proportions of α-helix, β-sheet, β-turn and random coil percentages were calculated using Peak Fit v4.12 software by selecting the amide I band in the spectral region of 1600–1700 cm^−1^.

### 2.10. Microscopy

The microstructure and morphology of gel samples were analyzed using a modified method described by Zhao et al. [18]. Gel samples were cut into small pieces (2 mm × 2 mm × 2 mm), fixed with 2.5% glutaraldehyde solution (pH 6.8), and stored at 4 °C. An ethanol solution with volume fractions of 50%, 70% and 90% was dehydrated once, anhydrous ethanol was dehydrated three times, then the mixture (the volume ratio of anhydrous ethanol to tert-butanol was 1:1) and the pure tert-butanol were replaced once, each time for 15 min. After freeze-drying treatment, a TA scanning electron microscope (SEM) (Zeiss Suppra-55, Jena, Germany) was used at a voltage of 10 kV and a magnification of 1000 times. At least three photographs were taken for each sample.

### 2.11. Statistical Analyses

Data were presented as mean ± standard deviation, and all experiments were conducted in triplicate under identical conditions. Statistical analyses of data were achieved using statistical package for social sciences (SPSS version 26, IBM, Armonk, NY, USA). Analysis of variance (ANOVA) was utilized to evaluate the statistical significance between all of the data, and Tukey’s b(K) test was applied to assess significant differences at a level of *p* < 0.05. Origin 2018 (Origin Lab Co.) was used for data visualization, and a heat map was generated in conjunction with Pearson’s correlation coefficient (r). 

## 3. Results and Discussion

### 3.1. Water-Holding Capacity

**Figure 1:**
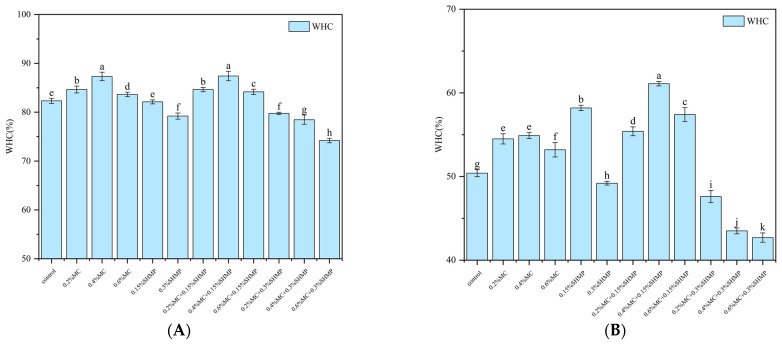
Effects of MC and SHMP on the WHC of SPI gels before (**A**) and after freezing (**B**). Different letters of the same indicator are significantly different (*p* < 0.05).

Water-holding capacity (WHC) is an important indicator of the protein–water interaction within a protein gel. In this study, SPI gel with higher water-holding capacity has better texture. As shown in Figure 1, a significant decrease in the WHC of SPI gels occurred due to freezing treatment. Complementing the findings from scanning electron microscopy (SEM) analysis, this phenomenon can be attributed to the susceptibility of the gel network to disruption caused by ice crystal formation during freezing, resulting in gel dehydration, shrinkage and a decline in functional properties. Notably, the freezing process exposes hydrophobic regions on the surface of SPI, further contributing to protein denaturation [19].

Methylcellulose (MC) significantly enhanced the WHC of SPI gels, which exhibited an initial increase followed by a decrease in WHC within the range of 0.2% MC to 0.6% MC. This suggests that MC, within a certain concentration range, can elevate WHC in SPI gels. It is speculated that with an increase in MC concentration, the reduced distance between MC molecules enhanced collision probability, and MC interacted with macromolecular proteins through intermolecular hydrogen bonding, forming a denser network structure with the formation of new cross-linkages [20]. Additionally, MC molecules possess substantial steric hindrance, which prevented excessive protein cross-linking and promoted the formation of a uniform and compact gel structure [21]. The best WHC was exhibited by the 0.4% MC-SPI gel (54.3%). However, the WHC of freeze-treated SPI gels supplemented with MC decreased significantly, with a notable decrease to 83.64% when the MC concentration reached 0.6%. In these cases, protein deposition occurred, indicating that a high concentration of MC can adversely affect the binding capacity of protein with water. Gao et al. [22] noted that excessive concentration of polysaccharides can affect WHC of heat-induced protein gels.

The effect of SHMP on improving the WHC of SPI gels was less pronounced, but it effectively led to a reduction in WHC under freezing condition. As depicted in Figure 1, the addition of SHMP increased WHC in SPI gels by 0.3%. However, following freezing treatment, 0.15% SHMP led to a more substantial increase in WHC compared to the unfrozen samples, thereby surpassing the WHC of freezing-treated SPI gels supplemented with MC. This observation is consistent with the gel texture result, which is attributed to the antifreeze properties of phosphates. This phenomenon stabilizes protein pH at neutral levels and forms a layer of bound water molecules on the protein surface, thereby protecting the protein structure and reducing protein freeze denaturation during the freezing process [14]. Furthermore, SHMP, with its high degree of polymerization and cyclic molecular structure [23], has limited penetration ability into the interior of the protein gel, resulting in relatively light cross-linking between SHMP and the protein gel [24]. This also accounts for the less pronounced improvement in WHC of SHMP/SPI gels before freezing. The subsequent addition of 0.3% SHMP concentration led to a significant reduction in WHC. There was a sharp decrease in WHC to 79.21% before freezing, which dropped to 48.4% after freezing. As SHMP is weakly acidic, the addition of a high concentration of SHMP altered the gel’s pH, as evidenced by the turbidity and particle size results, indicating structural disruption of the 0.3% SHMP-SPI gel. This is possibly due to a high concentration of SHMP which caused the extensive aggregation of SPI particles, thereby hindering SPI cross-linking during gelation. Wang et al. [25] suggested that phosphates can induce the self-aggregation of SPI and alter the protein structure and aggregation behavior of SPI.

The combined action of MC and SHMP on SPI gels exhibited a strong synergistic effect. In Figure 1, the WHC of the 0.4% MC-SPI/0.15% SHMP gel was 88.6%, which decreased to 67.3% after freezing. This was significantly higher than the gels of MC-SPI and SHMP-SPI (*p* < 0.05), emphasizing the role of phosphate groups combined with MC in strengthening the formation of the gel network. Similar findings were reported by Phanngam et al. [26], who observed that adding sodium tripolyphosphate to gelatin promoted gel network formation, enhanced gel strength, suppressed polysaccharide deposition and reduced electrostatic repulsion between aggregates.

### 3.2. Low-Field Nuclear Magnetic Resonance

**Figure 2:**
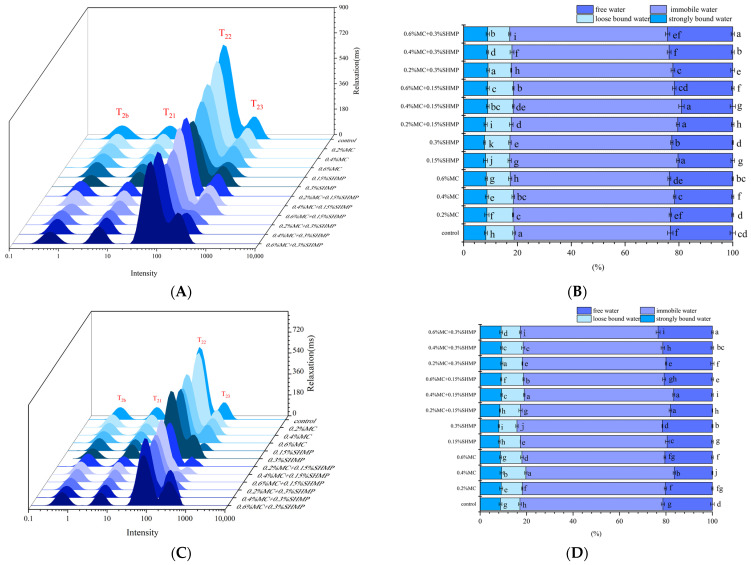
Relaxation distribution curves of unfrozen (**A**) and frozen (**C**) SPI gels and spin relaxation time ratios of unfrozen (**B**) and frozen (**D**) SPI gels. Different letters of the same indicator are significantly different (*p* < 0.05).

The distribution characteristics of water molecules in the gel, measured through T_2_ relaxation time, are an important method for studying the correlation between water molecules and proteins. Based on T_2_ relaxation time, water molecules in the gel can be classified into four types: tightly bound water, reflecting water tightly bound within the macromolecule (0.1 ms < T_2b_ < 1 ms); weakly bound water, indicating water bound within the macromolecule but less tightly (1 ms < T_21_ < 10 ms); immobile water, showing water bound within the macromolecule but not tightly (10 ms < T_22_ < 100 ms); and free water, representing water easily lost from the gel (100 ms < T_23_ < 1000 ms). As shown in Figure 2, freezing caused an increase in the proportion of free water in the SPI gel. Freezing led to the redistribution of water, causing disruption of the gel network structure and a reduction in its water-binding capacity. The e‘xpansion’ of the gel pore structure due to ice crystal formation during freezing promoted an increase in water molecules trapped within the gel structure, leading to a higher proportion of T_23_ (free water) [27]. Additionally, freezing directly results in the unfolding of the protein’s three-dimensional structure [28], which reduces the amount of water bound to the protein through non-covalent interactions. Therefore, there is a decrease in the proportion of T_22_ (immobile water) and an increase in the proportions of T_21_ and T_23_ after freezing.

The addition of MC resulted in a tighter binding of SPI with water, leading to significant reduction in free water content (*p* < 0.05). Water situated between protein gaps (T_22_) is influenced by MC, shifting towards the interior of the protein (T_2b_, T_21_). As a neutral polysaccharide, MC decreases the melting enthalpy, and the environmental entropy change encourages the binding of the gel with T_23_ (free water) [29]. 

Consequently, the content of T_22_ (immobile water) in the gel decreased, which provides further explanation for the increase in WHC after the addition of MC. On the other hand, after freezing, the proportion of free water in SPI gel with 0.4% MC remained lower than that in the blank gel, indicating an effective role in inhibiting an increase in free water content in the gel. This effect is related to the formation of a dense gel network through hydrophobic interactions and disulfide bonds. A robust gel network can retain water and reduce the free movement of water [19]. The higher proportion of weakly bound water in 0.4% MC-SPI gel further supports this idea. Previous research has also demonstrated cellulose ethers form gels with excellent water barrier properties and mechanical performance [30].

In Figure 2D, SHMP improved the proportion of bound water in SPI gel after freezing and reduced the proportion of free water. Furthermore, after freezing, the content of T_23_ (free water) in 0.15% SHMP-SPI gel was smaller than that in both MC-SPI and blank SPI gels (*p* < 0.05). This suggests that SHMP enhanced the water adsorption capacity of SPI gel after freezing. Zhu et al., found that the presence of AMP (Adenosine monophosphate) can increase the space between myofibrils in muscle, stimulate the interaction between immobile water and the filaments, inhibit the aggregation/denaturation of protein induced by freezing, and increase the water-holding capacity and mechanical properties of frozen-stored surimi [31]. Phosphates in a protein environment act as a buffer, which maintains the electrostatic attraction between protein molecules and water molecules. Additionally, phosphates can chelate cations within soy proteins [32], thereby creating space for protein-bound water molecules. This exposes negatively charged groups that firmly bind with water through non-covalent interactions, thereby reducing the content of free water in the gel [32]. The enhancing effect of phosphates on pH and ionic strength should not be overlooked, as it is beneficial in forming a solid network structure [13]. Furthermore, certain groups within protein molecules can react with hydroxyl groups induced by phosphates, thereby reducing the aggregation denaturation that arises due to functional group reactions between protein molecules [33].

In Figure 2D, MC and SHMP exhibited a strong synergistic effect on improving the texture of the SPI gel, with the 0.4% MC-SPI/0.15% SHMP gel having the lowest free water content before and after freezing. Phosphate groups are hydrophilic groups. The introduction of phosphate groups can enhance the water solubility of polysaccharides, further strengthening the gel network’s strength. Song et al. [34] suggested that phosphate groups can modify the water solubility of polysaccharides. However, this synergistic effect can be disrupted by high concentrations of MC or SHMP, leading to a gradual increase in the proportion of free water (*p* < 0.05). This indicates that during the freezing process, as moisture froze, available water for stabilizing protein structures steadily decreased, resulting in the disruption of the gel structure. Simultaneously, as the concentration of SHMP within the system increased, competition for water by inorganic ions led to a salting-out effect, which further destabilized the gel network [35].

### 3.3. Texture Profile Analysis (TPA)

**Figure 3:**
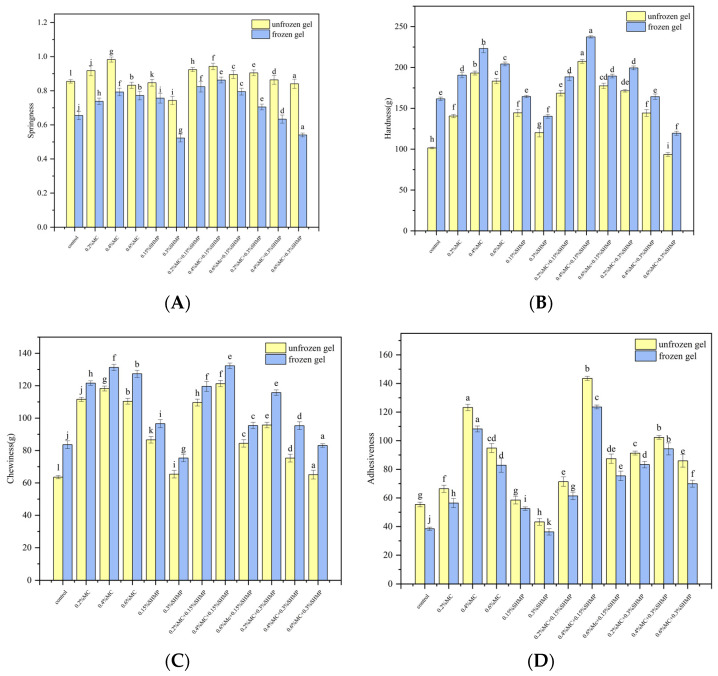
The effects of MC and SHMP on the springiness (**A**), hardness (**B**), chewiness (**C**) and adhesiveness (**D**) of unfrozen and frozen SPI gels. Different letters of the same indicator are significantly different (*p* < 0.05).

The textural properties of a gel play a crucial role in its quality, with hardness, springiness, chewiness and adhesiveness being important parameters for the textural characteristics of the gel [36]. Under the same treatment conditions, the texture of the gel in this study is better with high hardness, strong springiness, chewiness and adhesiveness. As shown in Figure 3, freezing increased the hardness and chewiness of the gel, while adhesiveness and springiness generally decreased.

Freezing also led to dehydration due to the narrowing of the distances between the three-dimensional networks within the gel’s structure, resulting in a denser colloidal structure and smaller spaces between the gel’s structural components. This contributed to an increase in gel hardness. However, the disruption of the gel network also significantly affected the gel matrix’s ability to retain water, leading to a decrease in springiness [27]. Furthermore, the primary forces maintaining gel-bonding networks are hydrogen bonds and van der Waals forces. After freezing, the available free hydroxyl groups for hydrogen bonding decreased, leading to a reduction in adhesiveness [18].

Figure 3 demonstrated that low concentrations of MC enhanced the strength of the SPI gel, but the inhibitory effect on the reduction of the frozen SPI gel was not significant. When the amount of MC added was in the range of 0.2% to 0.4%, the hardness, chewiness, adhesiveness and springiness of SPI gel all increased with increasing MC concentration. Due to the poor solubility of MC [37], its incorporation into the SPI gel network resulted in phase separation and mutual repulsion between MC and SPI, thereby creating a multiphase structure in the gel system. This led to steric hindrance and enhanced effective SPI concentration [38], thereby improving the gel’s strength. However, after freezing, the hardness, chewiness, adhesiveness and springiness of the SPI gel increased, while the adhesiveness and springiness generally decreased. The degree of the decrease was nearly identical to that of the blank gel (*p* < 0.05). MC primarily enhanced the network structure of the SPI gel through its thickening effect. However, post-freezing dehydration altered water distribution within the gel, resulting in irreversible gel reduction. This indicated that the addition of MC had an insignificant effect on inhibiting the reduction of the gel texture. When 0.6% MC was added, the strength of the SPI gel weakened, resulting in decreased hardness, chewiness, adhesiveness and springiness. The gel system’s structure transitioned from a dense and uniform continuous gel to a granular gel structure. The complex system’s network structure was disrupted, and SPI within the gel aggregated into clustered particles, thereby creating increased gaps between molecules. This was reflected in the reduced gel texture strength and weakened solid rheological characteristics [39]. Singh suggested that the addition of a significant amount of cellulose ether increases the aggregation of particles, thereby weakening the mechanical performance of the system [40].

Figure 3A,D illustrated that SHMP had an insignificant effect on the gel strength of the SPI gel. However, moderate amounts of SHMP significantly enhanced the gel’s freeze–thaw stability. Both the springiness and adhesiveness of SPI/SHMP gels decreased slightly before and after freezing (*p* < 0.05). This was because SHMP had a longer chain length and a higher degree of polymerization, resulting in a weaker gel strength due to less pronounced gelation with SPI [41]. However, after freezing, some of SHMP’s phosphate groups bound to free water and unfrozen water within the gel, leading to a decrease in the freezing point of the co-crystallization of some free and unfrozen water [36]. This reduction in ice crystal formation inhibited the enlargement of voids caused by ice crystals and prevented SPI aggregation. This resulted in improved texture characteristics of the gel, which reflected an increase in gel elasticity and adhesiveness. The addition of high concentrations of SHMP can disrupt the texture of the SPI gel. When 0.3% SHMP was added, the strength of SHMP/SPI gels decreased. Excessive SHMP not only caused SPI to undergo salting-out, which promoted irregular protein aggregation [17], but also disrupted the three-dimensional spatial structure of the SPI gel network formed by adjacent SPI molecules through forces such as disulfide bonds, hydrogen bonds, and hydrophobic interactions.

In Figure 3, MC and SHMP exhibited a strong synergistic effect on frozen SPI gel. The addition of 0.4% MC and 0.15% SHMP resulted in the highest gel hardness, springiness, chewiness and adhesiveness, with the best-maintained texture characteristics after freezing. The thickening effect possibly induced by MC further strengthened the improvement in the freeze–thaw stability of the gel network by SHMP. Tang et al. [17] suggested that the addition of salt ions can enhance the mechanical properties of polysaccharide gels and improve their microstructure.

### 3.4. Rheological Properties

**Figure 4:**
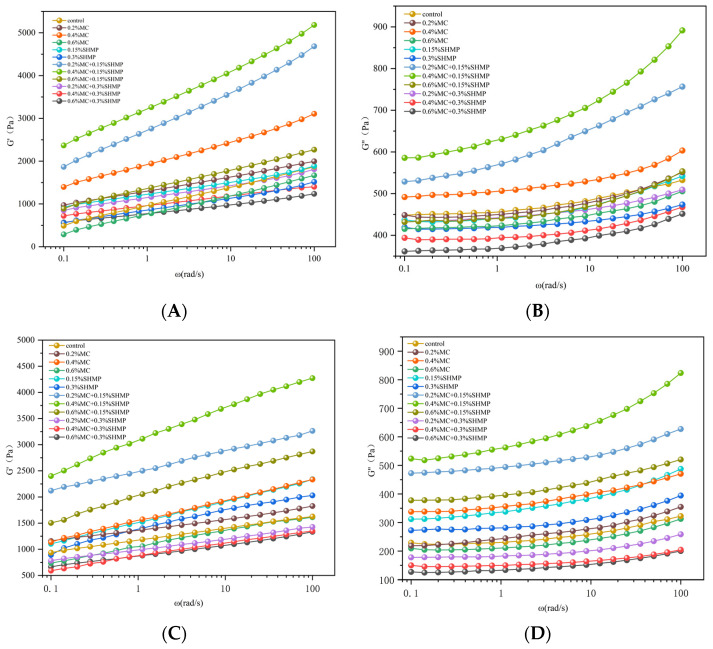
Effects of MC and SHMP on the storage modulus (**A**,**C**) and loss modulus (**B**,**D**) of unfrozen (**A**,**B**) and frozen (**C**,**D**) SPI gels.

The dynamic viscoelastic properties of a protein gel system can reflect the three-dimensional spatial network structure of the gel, where the storage modulus, also known as the elastic modulus, reflects the material’s elasticity, and the loss modulus, also known as the viscous modulus, reflects the material’s viscosity. MC can increase the storage modulus and loss modulus of the SPI gel, but freezing treatment significantly reduces both storage and loss moduli. As shown in Figure 4, G″ was consistently lower than G′, indicating that the SPI gel with added MC primarily exhibited stronger elastic properties [32].

SPI gels with 0.2% MC and 0.4% MC exhibited higher G’ and G″ compared to the blank gel (*p* < 0.05). This was because electrostatic interactions gradually exposed the charges encapsulated within SPI molecules, which then combined with the free negative charges in MC, thereby forming a uniform, stable network structure that was enhanced by hydrogen bonding [14]. For MC-added SPI gels subjected to freezing, both G’ and G″ significantly decreased, indicating that improvement in gel freeze–thaw stability due to MC was not substantial. This is highly related to the gel’s freeze–thaw stability [42], suggesting that the network structure formed by MC did not enhance water mobility within the gel. Excessive MC addition can reduce the storage modulus and loss modulus of SPI gels. At 0.6% MC, G’ of the system significantly decreased and was lower than that of the blank gel. When the polysaccharide addition is relatively high, it can lead to the appearance of aggregates, disrupting the gel structure [43]. It is also possible that with an increase in MC concentration, intermolecular interactions between SPI and MC in the gel system were further strengthened, thereby reducing the availability of total functional groups and decreasing G″, which mainly relies on hydrogen bonding forces [32].

The addition of SHMP increased the G’ and G″ of the SPI gel. However, when subjected to freezing, the SPI gel with SHMP consistently exhibited higher G’ and G″ than the blank gel. In the 0.15% SHMP-SPI gel, the addition of phosphate into the protein increased the solubility. This implies that more protein molecules were involved in the formation of protein–water hydrogen bonds during the gelation process, thereby increasing the interactions between molecules, and leading to an increase in storage modulus. Furthermore, Liu et al. found that phosphate treatment in refrigerated foods can effectively maintain a stable pH environment, improving food texture [44]. Wu et al. found that sodium pyrophosphate can increase the G’ value of frozen-stored surimi, indicating that phosphate compounds can inhibit the denaturation of myofibrillar protein during freezing storage, and alleviate the decline of gelling ability [45]. The G’ and G” values of frozen-stored surimi added with AMP were higher than those of the control group, and the difference was more obvious with the extension of frozen storage time [31]. On the other hand, excessive SHMP inhibited the rheological properties of the SPI gel. The G′ of the 0.3% SHMP-SPI gel significantly decreased. High concentrations of phosphate increased the charge repulsion between SPI gel particles, altered the pH properties of the gel environment and hindered the cross-linking of SPI particles during the gelation process. This led to the formation of more pores and a poorer gel structure. 

As shown in Figure 4, gels with MC and SHMP exhibited significant frequency dependency. Additionally, SPI gel with 0.4% MC and 0.15% SHMP showed G’ and G‘‘ values higher than those of gels with either MC-SPI or SPI/SHMP alone. This suggests a synergistic effect between MC and SHMP. Studies have explained that this frequency dependency could be related to the formation and breaking of different colloid bonds [46]. It is hypothesized that, compared to the single-system SPI gel, the addition of MC and SHMP may lead to more bond formation and the breaking of the gel structure due to the synergistic effects of phosphate and polysaccharides. The analysis of intermolecular forces and particle size also supports this hypothesis.

### 3.5. Protein Secondary Structures

**Figure 5:**
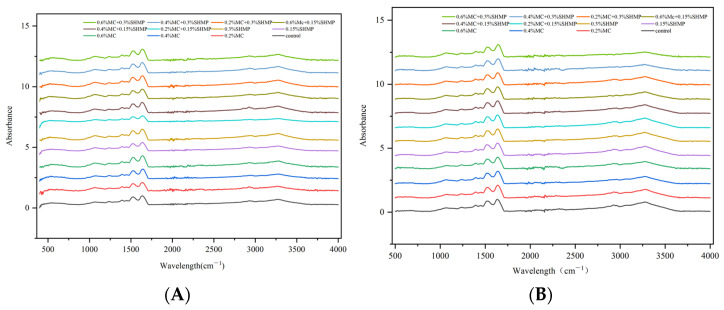
Fourier transform infrared spectroscopy (FTIR) of gelatinous protein samples with different MC and SHMP before (**A**) and after freezing (**B**).

The primary characteristic spectral bands of proteins are primarily associated with the infrared absorption of amide I, where the stretching vibrations of C=O or C=N bonds are predominant. Specifically, the range of 1610–1630 cm^−1^ is attributed to β-sheet structures, 1640–1660 cm^−1^ to unordered coil structures, 1661–1680 cm^−1^ to α-helix structures, and 1681–1700 cm^−1^ to β-turn structures [42]. Utilizing Fourier self-deconvolution for the amide I band and subsequently fitting Gaussian curves to the amide I band on the infrared spectra, more detailed information regarding the secondary structure were obtained, as presented in Table 1 and Figure 5. A moderate amount of MC enhanced the proportion of flexible structures within the SPI, thereby transforming protein molecules from a disordered state to an ordered state. However, MC did not effectively suppress the decrease in the proportion of ordered structures caused by freezing. SPI gels containing 0.2% and 0.4% MC showed an increase in α-helix and β-sheet structures, indicating alterations in the secondary structure of SPI. The addition of polysaccharides promoted the formation of intermolecular hydrogen bonds between carbonyl and amino groups, leading to an increase in α-helix content [20].

Upon freezing, the α-helix and β-sheet contents of blank SPI gels decreased by 1.984 and 1.383, respectively. SPI gels with 0.4% MC exhibited reductions of 3.31 and 1.054 in α-helix and β-sheet contents, respectively. This suggests that MC cannot effectively inhibit the alteration of protein secondary structure. It is hypothesized that MC primarily improved the performance of protein gels by constructing a dense gel network. However, when protein aggregation occurred on a large scale, the gel network was disrupted upon protein re-aggregation. Excessive MC diminished the proportion of flexible structures within the SPI. At an MC content of 0.6%, the contents of α-helix and β-sheet structures decreased, while β-turn and unordered coil contents increased. Liu et al. [47] also concurred that a high concentration of polysaccharides can disrupt the thermally induced orderly structure of SPI. Furthermore, in the vicinity of the 3300 cm^−1^ (N-H stretching vibration) region, there was a reduction in the amidine peak. This reduction implied that the excessive addition of MC led to the saturation of electrostatic interactions between SPI and MC, consequently weakening the formation of N-H bonds within the gel system. As a result, the N-H peak in compounds containing amino groups within the protein, whether as free or complexed amino groups, diminishes.

The denaturation and aggregation of proteins in a gel can be reflected by changes in the protein’s secondary structure [33]. As shown in Table 1, compared to the gel without SHMP, the addition of 0.15% SHMP to the SPI gel resulted in a lower decrease in the proportion of α-helix and β-sheet structures before and after freezing. This indicates that the addition of SHMP not only promoted the formation of ordered protein structures but also assisted in maintaining these ordered structures [13]. During the freezing process, protein molecules aggregated excessively, leading to the formation of heterogeneities and larger protein aggregates. These protein aggregates further embedded functional groups internally, leading to a reduction in non-covalent cross-linking [48]. However, SHMP enhanced non-covalent cross-linking through ion coupling and induced the gel to form more hydrogen bonds due to hydrophilic phosphate groups, thereby reducing protein aggregation. This effectively protected the non-covalent cross-linking of the gel. Excessive SHMP (0.3%) disrupted the ordered protein structure of SPI, resulting in a significant decrease in α-helices and β-sheets. 

On the other hand, it was observed that in the 0.4% MC-SPI/0.15% SHMP gel, α-helix content was significantly higher compared to MC-SPI, SHMP/SPI and the blank gel. Based on the rheological results, it can be inferred that the addition of SHMP affected the interactions between MC-SPI, leading to change in the original protein’s secondary structure. Zhu et al. [49] found that under conditions involving the addition of salt ions, polysaccharides can bind to positively charged regions on the protein surface through electrostatic interactions, promoting protein unfolding and altering the protein’s secondary structure. However, this synergistic effect becomes ineffective at high concentrations of SHMP or MC. In the cases of 0.6% MC-SPI/0.4% SHMP, 0.4% MC-SPI/0.4% SHMP and 0.2% MC-SPI/0.4% SHMP, there was a significant decrease in both α-helices and β-sheets. Lozinsky et al. [50] proposed that the increase in salt content enlarges the volume of unfrozen liquid in the system’s microphase regions, which results in a reduction in the concentration of polysaccharides within the unfrozen liquid microphase, thereby weakening the interactions between polysaccharide molecules.

### 3.6. Intermolecular Force

**Figure 6:**
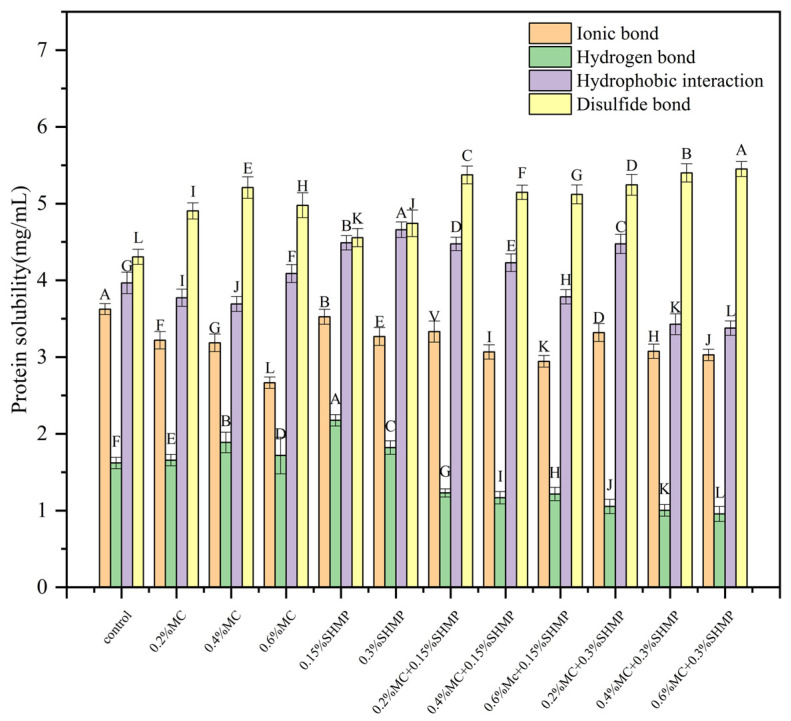
The effects of MC and SHMP on the interaction forces between frozen SPI gels. Different letters of the same indicator are significantly different (*p* < 0.05).

The transformation of intermolecular forces (hydrogen bonds, ionic bonds, hydrophobic interactions, and disulfide bonds) is generally considered the primary reason for changes in the properties of protein gels [51]. The addition of MC enhanced the proportion of hydrogen bonds in the SPI gel (Figure 6). This promoted the formation of a highly cross-linked, structurally dense network of soy protein. However, the strength of hydrophobic interactions initially decreased and then increased with the addition of MC. This was because MC is a low-substituted cellulose ether with a relatively uneven distribution of substituents, meaning there were regions on the same cellulose chain with high substitution and almost no substitution [37]. This resulted in significant steric hindrance with MC, thereby requiring a higher concentration to exhibit noticeable cross-linking effects.

Additionally, MC contains a large number of hydrophobic methyl groups. Therefore, as MC increased, hydrophobic interactions within the gel were enhanced. However, this phenomenon can lead to reduced protein stability [52], causing hydrogen bond breakage, the exposure of hydrophobic groups, hydrophobic aggregation, and the disruption of gel network structure formation. On the other hand, MC can enhance disulfide bond interactions in the protein gel. It is speculated that MC not only strengthened the cross-linking of SPI but filled the SPI gel matrix with the polysaccharide gel formed, thereby preventing the gel network from being disrupted and reducing protein aggregation. Tang et al. [53] provided similar conclusions, suggesting that cellulose ethers can enhance protein stability by strengthening intrachain and interchain disulfide bonds.

The gelation system in the frozen gel was relatively closed, and its structure was ordered [54]. The improvement in gel order can only be achieved through external factors. SHMP has a certain degree of acidity, and its addition causes the pH of the gel to deviate from the isoelectric point, leading to an increase in disulfide bond proportion and an improvement in the degree of the cross-linking of the protein [51]. Adding an appropriate amount of SHMP can increase the proportion of hydrogen bonds in the SPI gel, as shown in Figure 6. The hydrogen bond proportion in the 0.15% SHMP/SPI gel was higher than that in the blank gel. Combined with the TPA and WHC results, hydrophilic phosphate groups induced the formation of hydrogen bonds in the gel, leading to an increase in the hydrogen bond proportion. However, excessive amount of SHMP disrupted the hydrogen bond proportion in the SPI gel. In the 0.3% SHMP/SPI gel, the proportions of hydrogen bonds and disulfide bonds decreased significantly. High concentrations of phosphate disrupted the gel network, unraveled intermolecular hydrogen bonds, exposed hydrophobic groups and enhanced hydrophobic interactions. Liu et al. [55] held a similar view. It is speculated that negative charges and phosphate groups in the gel repelled each other. When phosphate salts were added to SPI, the initial equilibrium state was disrupted. This also promoted the extension of the protein structure, which was supported by the infrared results. During the gel freezing process, changes in internal intermolecular forces such as hydrogen bond breakage, the exposure of hydrophobic groups and disulfide bond content often occur [56]. Zhao et al. [18] studied the influence of freeze–thaw cycles on the properties of SPI gels and found that disulfide bonds contributed more to gel network formation than hydrogen bonds and hydrophobic interactions. Furthermore, due to the longer structural formula of SHMP and its lower dissociation degree, the degree of the cross-linking of protein molecules in the system was relatively low. This also explains the phenomenon that the improvement in the ion bond concentration of protein by SHMP was not significant compared to other phosphates [14].

When MC and SHMP acted together on the SPI gel, disulfide bonds increased significantly, while hydrogen bonds decreased significantly. This further indicates that the mutual cross-linking of MC-SPI/SHMP enhanced the formation of the gel network. The addition of SHMP led to changes in environmental acidity and alkalinity. Due to the irregular structure of MC, the distribution of electron clouds was uneven. Therefore, MC was highly responsive to environmental changes [57]. It is speculated that this is also the reason for the change in the disulfide bond proportion in MC-SPI/SHMP gel.

### 3.7. Microscopy

**Figure 7:**
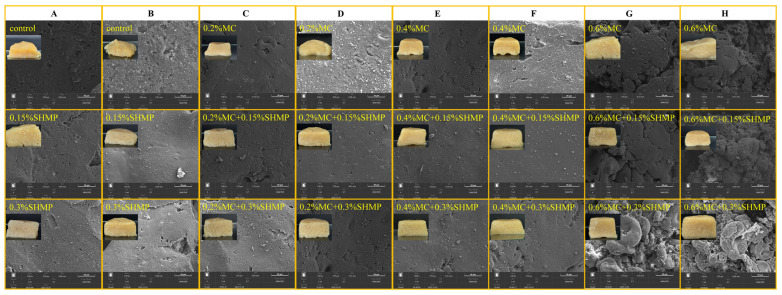
Scanning electron micrographs of SPI gels before (**A**,**C**,**E**,**G**) and after freezing (**B**,**D**,**F**,**H**), with MC and SHMP.

Scanning electron microscopy (SEM) provides visual insight into the microstructure of protein gels. SPI gels with the addition of MC and SHMP revealed that as the MC concentration increased from 0.2% to 0.4%, the gel pores became smaller, and no significant phase separation was observed (Figure 7). This could be related to MC’s thickening effect. Furthermore, as the MC concentration increased from 0.4% to 0.6%, the gel structure was disrupted, and aggregation occurred, indicating increased heterogeneity in the gel system. This may be because the higher concentration of polysaccharides facilitated incomplete gel separation, resulting in non-uniformity in the gel, which is consistent with the texture results. This can also be observed from the physical picture of gel sample. Li et al. [58] and others have also suggested that high concentrations of polysaccharides can lead to incomplete gel separation. Another possibility is that the addition of high concentrations of polysaccharides causes protein aggregation, which alters protein–polysaccharide interactions and disrupts the gel network structure [3]. Excessive MC can disrupt the equilibrium of mixed gels, leading to gel structure disruption [59]. Interestingly, as with the previous experimental results for other indicators, the protective effect of MC on frozen SPI gels was not significant.

As shown in Figure 7, the addition of SHMP had a mild impact on the structure of SPI gels. However, SHMP had a positive effect on the frozen SPI gel, resulting in fewer pores and a more homogeneous gel structure. Since phosphate salts can potentially interrupt phase separation before gelation, leading to faster gelation [60], the disruption of phase separation affects incomplete separation within the gel system, making the gel system more uniform [61]. This suggests that SHMP can enhance the cross-linking interactions between proteins, thereby optimizing the gel network formed among the proteins. In Figure 7, MC-SPI/SHMP gels exhibited the tightest and most uniform microstructure, while the gel could also maintain its apparent shape after freezing, indicating a significant synergistic effect between MC and SHMP in improving the microstructure of SPI gels. Memic et al. [62] had a similar view, suggesting that the addition of salt ions can lower the freezing point temperature of the precursor solution, inhibit ice crystal formation, suppress freeze concentration effects, and ultimately improve gel properties.

### 3.8. Heat Map

**Figure 8:**
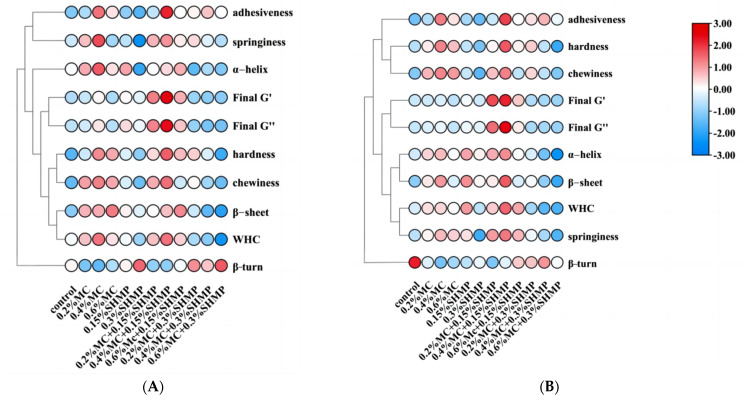
Heat maps based on SPI gel properties with different concentrations of MC and SHMP before (**A**) and after freezing (**B**). Hot tones indicate high index values and cool tones indicate low index values.

To visually represent the impact of MC and SHMP on the properties of mixed gels, a heatmap was generated to display the trends in each index. In the heatmap, colors represent the relative indices of each target characteristic, with red indicating high values and blue indicating low values (Figure 8).

The samples are divided into Group I (MC-SPI gels), Group II (SHMP/SPI gels), and Group III (MC-SPI/SHMP gels). The most significant changes in all measured indices in the first group were elicited by 0.4% MC-SPI, with changes more concentrated in TPA and rheology. In the second group, the most significant changes in all test indices were elicited by 0.15% SHMP/SP. In the third group, almost all indices changed significantly for 0.4% MC-SPI/0.15% SHMP compared to other experimental groups. Overall, before and after freezing, both MC and SHMP appeared to improve the SPI gels. MC is more inclined to influence the rheological and texture characteristics of gel, while SHMP tends to promote protein aggregation. The color changes in the heatmap were more pronounced after adding SHMP, indicating that SHMP can enhance the freezing stability of the gel to some extent. The results for 0.4% MC-SPI/0.15% SHMP also suggest that there is some form of synergistic interaction between MC-SPI and SHMP, leading to a significant improvement in gel strength.

**Figure 9:**
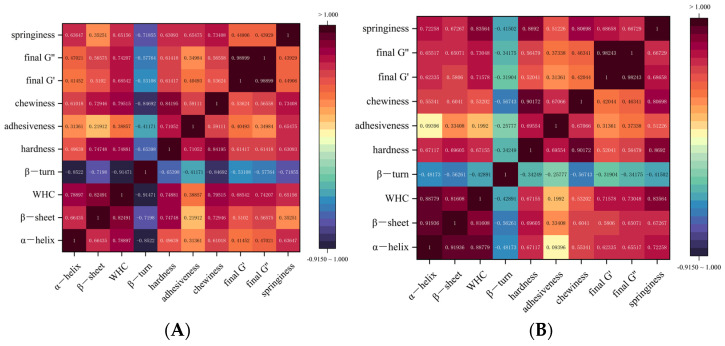
Heat map of the correlation between the rheological and structural parameters of MC-SHMP SPI gels before (**A**) and after freezing (**B**) and the protein structure.

Pearson correlation coefficient (r) was used to establish the correlations between the rheological and structural parameters of SPI gels, as well as the protein structure, before and after freezing with MC and SHMP; r-values vary between −1 and 1 and are color-coded to indicate the degree of correlation [63]. Based on the trend of gel properties with changes in α-helix and β-sheet content, as shown in Figure 9, there was a significant positive correlation (*p* < 0.05) between α-helix, β-sheet, and elasticity, viscosity, Final G″, Final G’, and a highly significant positive correlation (*p* < 0.01) between α-helix, β-sheet, and WHC, hardness, and chewiness. When assessed after freezing, the correlation between α-helix, β-sheet, and TPA parameters decreased slightly, while the correlation with rheological parameters increased. This suggests that changes in the SPI structure directly affected gel rheology and structural parameters. Therefore, enhancing gel freeze stability can be achieved by inhibiting SPI’s structural changes and reducing protein aggregation through the addition of different concentrations of MC and SHMP.

## 4. Conclusions

The effect of different concentrations of MC and SHMP on the properties of SPI gel before and after freezing were studied. The addition of MC enhanced the gel network and improved the gel strength of protein, but the freezing stability was not significantly improved. The addition of SHMP did not significantly improve the texture and network structure of the gel, but significantly improved the stability of the frozen protein, and increased the proportion of the α-helix and β-sheet after freezing, which was conducive for the gelation of the protein. MC and SHMP had a synergistic effect with the SPI gel system. This is mainly due to the fact that the phosphoric acid groups provided by SHMP solvated with water, thickening MC and increasing its water solubility. Compared with the blank control, the addition of 0.4% Mc–0.15% SHMP gel elicited the highest strength and energy storage modulus, and the optimal microstructure was obtained mainly through stable intermolecular interaction. Methyl substituents provided by MC promoted the release of the ionizable groups of SPI, resulting in the further aggregation of negatively charged SHMP groups on SPI. Hydrogen bonding and ionic interaction further inhibited the aggregation and denaturation of protein molecules. The regulation of MC and SHMP can significantly improve the structure and stability of protein gel, and inhibit the quality reduction caused by freezing, which provides practical guidance for the application of frozen SPI gel.

## Figures and Tables

**Table 1:** Relative content of the secondary structure of SPI gels before and after freezing with different MC and SHMP. Different letters of the same indicator are significantly different (*p* < 0.05).

Sample Addition	α-Helix		β-Sheet		β-Corner		Random Coil	
	Unfrozen Gel	Frozen Gel	Unfrozen Gel	Frozen Gel	Unfrozen Gel	Frozen Gel	Unfrozen Gel	Frozen Gel
control	25.32 h	23.34 j	34.27 j	32.88 k	18.42 f	22.45 a	21.98 c	21.32 d
0.2% MC	27.51 d	25.57 e	37.77 d	35.44 d	14.42 k	20.21 h	17.31 j	18.78 h
0.4% MC	29.49 b	26.18 d	37.88 c	36.82 c	13.73 l	19.32 l	20.90 g	17.67 j
0.6% MC	26.25 g	24.43 g	38.79 a	34.32 h	15.92 h	19.71 j	19.04 h	21.54 c
0.15% SHMP	27.65 c	26.76 b	36.71 f	36.97 b	18.46 e	19.93 i	17.18 k	16.33 k
0.3% SHMP	20.53 l	24.94 f	35.84 h	35.22 f	22.19 b	20.32 f	21.43 e	19.52 g
0.2% MC + 0.15% SHMP	30.56 a	26.56 c	36.56 g	35.42 e	15.11 i	19.39 k	17.77 i	18.62 i
0.4% MC + 0.15% SHMP	31.44 f	27.32 a	37.53 e	37.94 a	14.99 j	20.23 g	16.04 f	14.50 l
0.6% MC + 0.15% SHMP	27.38 e	24.38 h	38.56 b	34.29 i	17.78 g	20.95 d	16.29 l	20.37 f
0.2% MC + 0.3% SHMP	21.56 k	23.56 i	35.48 i	34.83 g	21.11 c	21.02 c	21.86 d	20.60 e
0.4% MC + 0.3% SHMP	23.44 i	20.98 k	33.45 k	32.98 j	19.61 d	21.42 b	23.50 a	24.61 b
0.6% MC + 0.3% SHMP	22.42 j	18.24 l	32.43 l	31.37 l	22.58 a	20.45 e	22.57 b	29.95 a

## Data Availability

The data presented in this study are available on request from the corresponding author. The data are not publicly available due to restrictions of privacy or ethical.
